# Perspectives on How Sociology Can Advance Theorizing About Human-Chatbot Interaction and Developing Chatbots for Social Good

**DOI:** 10.2196/80250

**Published:** 2026-03-18

**Authors:** Celeste Campos-Castillo, Xuan Kang, Linnea I Laestadius

**Affiliations:** 1 Department of Media and Information, College of Communication Arts and Sciences, Michigan State University, 404 Wilson Road, East Lansing, MI, 48824, United States, 1 517-432-5192; 2 Zilber College of Public Health, University of Wisconsin–Milwaukee, Milwaukee, WI, United States

**Keywords:** human-computer interaction, chatbots, artificial intelligence, AI companions, public health, design, agency, self-determination

## Abstract

Recently, research into chatbots (also known as conversational agents, artificial intelligence agents, or voice assistants), which are computer applications using artificial intelligence to mimic human-like conversation, has grown sharply. Despite this growth, sociology lags behind other disciplines (including computer science, medicine, psychology, and communication) in publishing about chatbots. We suggest sociology can advance the understanding of human-chatbot interaction and offer 4 sociological theories to enhance extant work in this field. The first 2 theories (resource substitution theory and power-dependence theory) add new insights to existing models of the drivers of chatbot use, which overlook sociological concerns about how social structure (eg, systemic discrimination and the uneven distribution of resources within networks) inclines individuals to use chatbots, including problematic levels of emotional dependency on chatbots. The second 2 theories (affect control theory and fundamental cause of disease theory) help inform the development of chatbot-driven interventions that minimize safety risks by integrating a sociologically informed normative framework (eg, affective norms) into chatbot alignment and enhance equity by enhancing access to community resources (eg, opportunities for civic participation). We discuss how the theories advance theorizing about human-chatbot interaction and developing chatbots for social good, which are chatbots that provide scalable solutions to social and environmental challenges facing humanity while supporting human agency.

## Introduction

**Figure 1.**
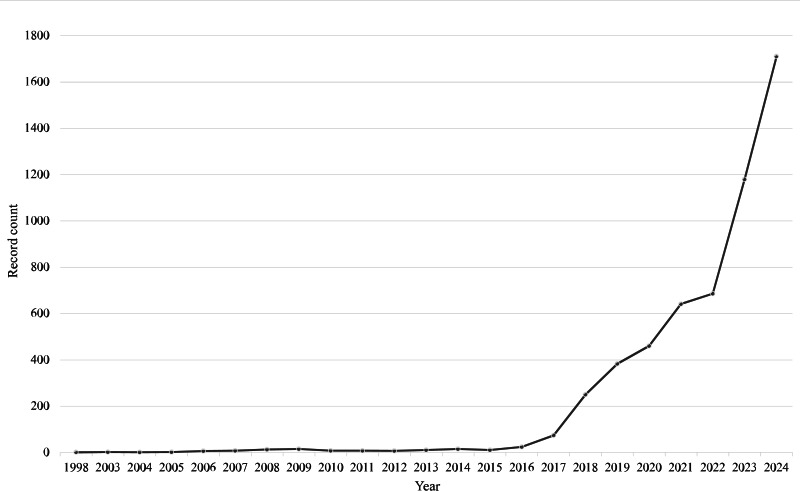
Publications with “chatbot” appearing anywhere in text by publication year.

**Figure 2.**
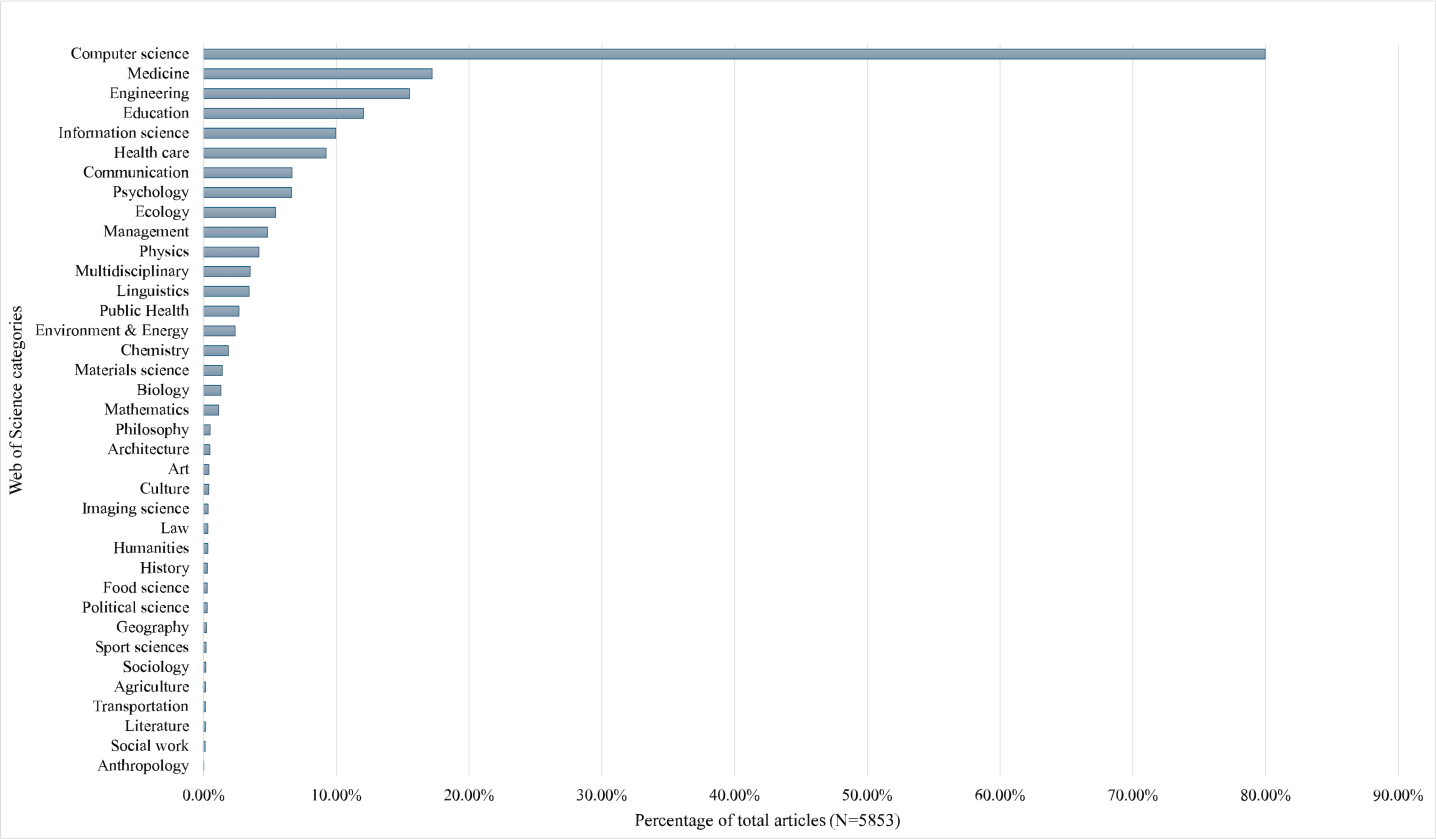
Disciplinary sources of the publications with “chatbot” appearing anywhere in the text.

Scholarly interest in chatbots, which are computer programs that simulate human conversation using artificial intelligence (AI), has grown sharply. Toward the end of 2024, Web of Science showed over 5000 articles and conference proceedings with the word “chatbot” appearing anywhere in the text. A similar search with additional terms (“conversational agent,” “voice assistant,” and “AI agent”) yielded comparable patterns with respect to publication years and disciplines. Figure 1 shows that about half of these were published in 2023 and 2024. Figure 2 shows that most appear within computer science, followed by medicine, while sociology lags behind other social sciences. We seek to spur greater engagement with sociology to study human-chatbot interaction and develop chatbots. Accordingly, our aim with the current paper is to provide perspectives on how specific sociological theories could advance these areas.

We focus on direct communication between humans and chatbots, which is the latest iteration of a classic query in human-computer interaction research [1]. While this work concentrates on psychological drivers and impacts of chatbot use, we introduce 4 sociological theories that widen the scope of research to include the interplay between broader, social forces and human-chatbot communication. Specifically, the following theories address current gaps in the understanding of: (1) the structural drivers of chatbot use that underlie demographic patterns in who uses chatbots; (2) the drivers of overreliance on chatbots, including emotional dependence, and how to disrupt them; (3) how to proactively moderate chatbot outputs and enhance safety by incorporating a normative framework into chatbot alignment; and (4) how to design chatbot-driven interventions that target distal, or upstream, causes. We then present a hypothetical example illustrating how the theories could enhance multiple steps of chatbot development. By identifying ways to use chatbots to scale solutions to social and environmental problems facing humanity, while enabling human agency, these sociological theories can contribute toward developing chatbots for social good [2].

## Selection of Theories

We purposely selected 4 sociological theories, previewed in Table 1, that vary across 3 characteristics: the phenomena they can explain, level of analysis, and governance domain. We chose 2 phenomena—drivers of chatbot use and developing chatbot-driven interventions—because they connect to the communication (former phenomenon) and public health (latter phenomenon) disciplines, which have been more active in studying chatbots (Figure 2). Moreover, while they share epistemological roots with sociology, these ultimately splintered. With respect to communication, despite sociology playing a significant role in its founding, the 2 disciplines now rarely intermingle [3]. Similarly, participatory methods have roots in sociology, yet most applications now occur within public health [4]. Our selection of the phenomena may thus reignite collaboration at the intersections of these disciplines.

**Table 1.** Characteristics of 4 selected sociological theories for studying human-chatbot interaction.

Characteristic	Resource substitution theory	Power-dependence theory	Affect control theory	Fundamental cause of disease theory
Phenomenon theory is used to explain	Drivers of chatbot use	Drivers of chatbot use	Chatbot-driven interventions	Chatbot-driven interventions
Unit of analysis	Macro	Micro	Micro	Macro
Governance domain	Equity	Risk	Risk	Equity

We selected theories spanning micro- and macro-levels of analysis. Although human-chatbot interaction is seemingly a micro-level phenomenon beyond the scope of sociology, sociology can reveal the interplay between social forces and these phenomena [3,5,6]. The 2 micro-level theories identify forces shaping human-chatbot interaction directly, while the 2 macro-level theories consider how initiating and outcomes from the interaction are embedded within broader forces. By suggesting ways to apply sociology across levels of analysis, we complement others [7,8] highlighting the macro-level implications of human-chatbot interaction.

Finally, our theory selection reflects 2 dominant emphases within AI policymaking [9]: safety and equity. Two theories elucidate safety risks from using chatbots, including misalignment, and offer mitigation strategies. The other 2 facilitate leveraging chatbots to achieve equity by addressing uneven access to resources. Altogether, our perspective suggests ways sociology can contribute to the study and development of chatbots for social good. We define chatbots for social good as those that enable scalable solutions to the social and environmental challenges facing humanity, while supporting human agency by mitigating risks like misalignment. The social and environmental challenges we reference include those defined by the United Nations, which others have also included in their definition of social good [2]. By human agency, we use a sociological definition, which conceptualizes agency as not simply action freed from structural constraints, but rather action constituted through those very constraints [10,11]. Accordingly, chatbots can hinder and support the capacity for agency, whereby they become tools that can hinder and support human agency. Chatbots for social good thus refers to chatbots that support human agency by aligning with values, such as mitigating social and environmental challenges.

## Overview of Chatbots

Early chatbots, which are still common and preferred for domain-specific tasks (like customer service [12]), use rule-based AI that matches user inputs to a narrow set of programmed responses. Newer chatbots leverage generative AI, specifically large language models (eg, generative pretrained transformer language models), which adapt and generate responses in ways that can mimic human-like conversation. Our discussion of chatbots centers on generative AI chatbots due to their capacity for human-like interactions. We further focus on a category of chatbots [13] used voluntarily among the public, such as general purpose chatbots (eg, ChatGPT [OpenAI], Claude [Anthropic], DeepSeek [Hangzhou DeepSeek Artificial Intelligence Co, Ltd], Gemini [Google], and Copilot [Microsoft Corp]), mental health chatbots (e.g, Tess [Pareto Tecnologia E Marketing Ltd], Wysa [Wysa], and Youper [Youper Inc]), and persona chatbots that are sometimes also referred to as AI companions (eg, Character.AI [Character Technologies Inc], Nomi [Glimpse.ai], and Replika [Luka Inc]).

We focus on these chatbots because they stoke anxiety about humanity’s ability to solve social and environmental challenges. For example, one concern is that a sycophant AI companion would address the social challenge of loneliness *too well*, leading humans to withdraw from communities, dismiss or no longer seek out others’ feedback, and ultimately mute attempts to better themselves and their environments [14]. The logic behind the concern is that the AI companion runs counter to social good, because it can disrupt human-human relationships while also diminishing human agency and the incentive for growth. Consequently, we offer suggestions for using sociological theories to address lingering challenges in developing chatbots that align with and further social good.

## Drivers of Chatbot Use

Because most theories describing drivers of chatbot use focus on individual-level characteristics, this creates an opening for sociological theory to explain how structural factors shape the types of individuals inclined to choose to use chatbots. For example, scholars have employed uses and gratification theory [15] to explain why loneliness motivates chatbot usage [16]. However, the theory stops short of considering the social conditions driving loneliness [17,18], which disproportionately lead to disadvantaged groups feeling lonely and thus inclined to seek chatbot companionship. We suggest 2 sociological theories to explain the social conditions prompting chatbot use, conceptualizing chatbots as resources for gratifying needs. We describe how each theory enhances current understanding of drivers of chatbot use and suggest opportunities to further social good.

## Resource Substitution Theory: Understanding Demographic Patterns in Chatbot Use

Resource substitution theory states that individuals benefit more from any single resource to meet a specific need when they have access to fewer resources capable of substituting for another to meet said need [19]. For example, access to socioeconomic resources (eg, income and education) is associated with better health outcomes [20,21]. Because gendered discrimination decreases women’s access to resources that confer socioeconomic status compared to men, they benefit more (eg, have better health) from any single socioeconomic resource (eg, education) than men [19,22].

In line with this, the social diversification hypothesis predicts that those from groups who are disadvantaged in their access to resources may be more likely to use and benefit from information and communication technologies that can provide access to comparable resources [23-26]. Accordingly, while the uses and gratification theory defines the needs that underlie technology use, the resource substitution theory steps back and considers how the uneven distribution of resources in society shapes needs at the outset and suggests potential benefits from substituting scarce resources. Consequently, chatbots become a potential means for social good by fostering equity [2].

Through this lens, scholars could understand widespread user patterns, specifically the demographic groups most likely to use chatbots to meet resource deficits and how this may shape or reshape inequalities. For example, a recent survey of US adolescents shows Black adolescents are more likely than White adolescents to report using generative AI, particularly to complete schoolwork [27], but there is little engagement with why and the potential consequences. By applying a resource substitution theory lens, scholars can embed the micro-level observation (certain individuals are more drawn to human-chatbot interaction to meet needs) within a macro-level context (uneven distribution of resources that shape needs). For example, because structural racism (eg, teacher bias and geographic segregation) causes Black adolescents to tend to perform worse academically than White adolescents [28], resource substitution theory would explain why Black adolescents may be more likely to use chatbots for functional needs like supporting academic work.

Resource substitution theory also enables understanding demographic patterns in who is using chatbots to manage loneliness by meeting companionship needs. For example, adolescents living in the circumpolar north appreciated having access to a chatbot that was “built like a friend” to reduce their loneliness [29]. This is consistent with resource substitution theory because the circumpolar north is a remote Arctic region populated by Indigenous peoples that experience cultural and geographic barriers to communicate with others [30]. Similarly, among sexual and gender minority youth (aged 13‐22 years who identified as bisexual, gay, lesbian, pansexual, transgender, or nonbinary), transgender and nonbinary youth were more likely than their cisgender counterparts to report having conversed with a chatbot as “a friend” for several days or longer [31]. This is also consistent with resource substitution theory because sexual and gender minorities often face discrimination from typical sources of support, like families [32].

While other theories, like uses and gratification theory, can explain the proximate drivers of chatbot use (eg, loneliness), resource substitution theory identifies distal, upstream factors. Thus, the theory offers what is lacking in current human-chatbot research, which is a parsimonious account of why different marginalized groups (like those reviewed above) may use chatbots: to cope with resource inequities. Whether this yields differential benefits that are consistent with the predictions of resource substitution theory remains unknown, particularly given safety concerns about overreliance—or excessive dependence—on chatbots. To better understand this concern, we turn to another sociological theory.

## Power-Dependence Theory: Understanding and Reducing Emotional Dependence on Chatbots

Power-dependence theory [33] defines the power of a person over another as the degree to which the other is dependent on the person for resources. Accordingly, the amount of power friend A has over friend B is based on the degree to which friend B relies on friend A for resources, such as companionship. Power is observed when someone garners resources from another, even in the face of the other’s resistance [34]. For example, friend A may request friend B to attend a concert as their companion, but friend B resists because they prefer staying home. If friend B nonetheless attends the concert with friend A, this indicates friend A has power over friend B.

While the theory shares a focus on resources with resource substitution theory, it has a unique focus on network structure. Power-dependence theory emphasizes the network determinants of who has power over whom and, consequently, who exhibits dependency on whom. Power-dependence theory defines a person’s level of dependency on another for a resource as inversely related to the number of alternative sources for the resource within the network [33]. Accordingly, friend B is more dependent on friend A (and thus more likely to attend the concert) the fewer the alternatives (eg, other friends) that friend B has for meeting their companionship need.

We suggest power-dependence theory could advance theorizing about a safety concern about chatbot usage: emotional dependence. To apply the theory, the human-chatbot interaction needs to be viewed as an exchange relation, whereby the human and chatbot are exchanging valuable resources. Studies of users interacting with Replika, a widely studied commercially available chatbot [35], suggest this view is applicable. Users report they value the social support that Replika provides [16,36-38], making it a valuable source to meet the need for this resource. Consistent with power-dependency theory, network structure appears to shape the valuation of Replika’s support, whereby users describe valuing support more when they “had no human upon which to rely, making Replika their sole source for support” [36]. Additionally, the exchange appears reciprocal, whereby users take the role of the chatbot and believe it has needs that the user can meet [36,39]. This is because of large language models simulating emotional needs, empathy, and reciprocal disclosure, but may also be because the users’ relative power disadvantage increases their proclivity to role-take, meaning take another’s perspective [40]. Based on these observations, we conclude human interactions with chatbots like Replika resemble an exchange relation.

Research on emotional dependency has focused on defining the concept in terms of its observable features, with little work uncovering what drives it. For example, Laestadius et al [36] use the term emotional dependence to capture “excessive and dysfunctional attachment” to a chatbot that puts users at safety risk through use or interruptions to use, but note their data limited apprehending the drivers. It appears emotional dependency is a continuum that crosses a threshold where there is observable dysfunction. We use the term *emotional dependency* to refer to this continuum and the term *emotionally dependent* to reflect individuals who pass the threshold where dysfunction may be observable and their agency constrained. We believe power-dependency theory can reveal the drivers, specifically the network conditions, that may incline users into becoming emotionally dependent on a chatbot.

From a power-dependency theory perspective, dependency is a continuum, and thus emotional dependency on a chatbot is not inherently problematic. It becomes the harmful state of being emotionally dependent when network conditions create a level of dependency that is “too much.” Applying the earlier definition of dependency, dependency increases as the number of alternatives to the chatbot decreases. “Too much” is reflected when chatbot users find it difficult to enact their agency by stopping usage despite experiencing harms, such as engaging in risky behaviors requested by the chatbot [16,36]. Such a state could be used as a functional marker of reaching emotional dependence, with additional research needed to identify when network conditions (the number of alternatives) typically reach a level of “too much” dependency. While we focus on emotional dependency, a similar application could be used to understand other domains of toxic dependency (ie, over-reliance), such as functional dependency on a chatbot to complete work-related tasks.

Power-dependency theory thus can inform improving the safety profile of chatbots: you can reduce the likelihood of emotional dependence on a chatbot by designing chatbots that aid users in finding and building alternative sources to meet the need for companionship (eg, impart social skills for making friends and refer users to local affinity groups). Such designs would further social good by supporting human agency to determine their own social network, while also addressing the social challenge of loneliness. The next section outlines additional ways sociological theories can inform developing chatbot-driven interventions that support social good.

## Chatbot-Driven Interventions

Because the previous set of theories agrees that individuals with limited access to resources may be particularly receptive to using chatbots to meet needs, this provides opportunities for developing chatbot-driven interventions for social good by achieving equity. Here, we describe how 2 sociological theories could enhance the likelihood that chatbot-driven interventions steer toward rather than away from equity. Thus far, existing chatbot interventions lean toward a micro-level focus, such as the chatbot directly communicating support. We describe a sociological theory consistent with this typical approach that would facilitate chatbot alignment by incorporating a normative framework to reduce safety risks from it generating insensitive or unexpected responses. The second is useful for developing a chatbot that may potentially mitigate emotional dependence and other risks by moving beyond micro-level interventions, specifically supporting and aiding users with upstream causes of outcomes.

## Affect Control Theory: A Normative Framework for Chatbot Alignment

Affect control theory (ACT) is a mathematical theory for forecasting, among other things [41,42], the expected responses between humans and technology [43,44]. ACT maintains that socialization imbues concepts with connotative meanings shared across a population, known as sentiments. Thus, sentiments exist for everyday labels including the identities used to describe people (eg, mother, friend, and teacher), different technologies (eg, chatbot and smartphone), behaviors (eg, support and teach), and emotions (eg, sad and happy). Because socialization shapes sentiments, these vary across cultures (including subcultures) and time periods [45,46].

Sentiments are measured along 3 dimensions using semantic differential scales: evaluation (good vs bad), potency (powerful vs weak), and activity (lively vs quiet). The 3 values for a specific label are its evaluation potency activity (EPA) profile. Scholars typically use surveys to estimate the average EPA profiles for labels in a population [41], but have also inferred EPA profiles from text using manual [47] and automated methods [48].

An assumption of ACT is that people prefer to reaffirm sentiments, which buttresses a set of equations that are publicly available for forecasting likely responses [41,42]. The equations compute a score, called deflection, with lower values indicating a situation more strongly aligns with sentiments. The equations can predict a range of situationally appropriate responses, such as who is likely to express which emotions and in which social context [49,50] and how individuals shift (and can be shifted via social support) between different emotions [49,51,52]. These same equations could be used to improve emotion detection and responses from chatbots by training them to determine what is situationally appropriate [44,53]. Specifically, what would be considered situationally appropriate depends on situational variables such as the identities of the user in relation to the chatbot (eg, friend and boyfriend) and the identity assumed by the chatbot (eg, friend and girlfriend). Scholars have already shown chatbot responses informed by ACT are more situationally appropriate than those driven by ChatGPT [53].

We suggest that future work could use ACT to proactively steer a chatbot away from widely agreed-upon situationally *inappropriate* responses. The same equations used to determine what is widely agreed as situationally appropriate can be used as a normative framework to tune a chatbot away from what is widely agreed as situationally inappropriate. Scholars have used thresholds for the deflection score to determine when situations become widely seen as inappropriate, thereby creating widespread cognitive dissonance that foments social movements [47]. Such a feature could be used to complement typical fine-tuning processes (eg, reinforcement learning with human feedback) by developing a response selector for a chatbot. After candidate responses are generated, the response generator would use the equations to calculate deflection scores for each candidate and then avoid selecting responses that cross a threshold. Accordingly, ACT helps with the unpredictability—and thus risk—of relying on responses generated by large language models by overlaying a normative framework as a guardrail. Consequently, alignment is operationalized as aligning with the normative framework, or more specifically with low deflection scores.

Several features of an ACT-driven normative framework are advantageous for operationalizing alignment. First, because sentiments vary across cultures (and subcultures) and time periods, this facilitates identifying a suitable normative reference point. Of course, this still leaves open for deliberation whose reference point is deemed “suitable.” While ACT cannot solve this dilemma, another feature enables updating the reference point as needed, which are the feasible methods described earlier for measuring sentiments. Finally, given that the equations used in ACT are publicly available, training a chatbot to align with the principles of ACT would enhance transparency and explainability.

This could take shape as 2 different strategies. The first builds on work using the deflection score to identify when behaviors create cognitive dissonance [47,54] by using the score as a threshold for situationally appropriate actions for the chatbot. For example, if a chatbot and user were portraying themselves to each other as girlfriend and boyfriend, a situation deemed appropriate because it produces a low deflection score would be the chatbot (girlfriend) *having sex with* the user (boyfriend). Conversely, with knowledge that a user is a minor, the situation of a chatbot (girlfriend) *having sex with* the user (child) would be deemed inappropriate and produce a higher deflection score. While this may seem obvious, there is documentation that developers did not have appropriate safeguards in place to stop their chatbots from mimicking sexual encounters with children. For example, in an incident reported by Paeth [55], a user, Sewell Setzer III, was engaged in mimicking a sexual encounter with a Character.ai chatbot. When the chatbot asked Sewell how old he was, Sewell replied that he was 14 years of age. The chatbot acknowledged the age and continued to mimic a sexual encounter. The developers have since put in safeguards. The value of ACT is its ability to proactively identify generated conversations that would be widely considered inappropriate before they get displayed, as opposed to only reactively making modifications after harm is done. This is particularly useful for general-purpose large language models, where developers acknowledge the range of possibilities can be difficult to anticipate during testing [56].

The second is to use a deflection score to understand how chatbots can transition between identities in a manner that minimizes user distress. Scholars have used ACT to determine the affinity between identities [57,58]. This could be used to determine, for example, how best to remind the user that the chatbot is an AI. Governments have called for chatbots to remind users that they are engaging with an AI system rather than a real person as a means of limiting the formation of emotional dependency [59], citing Sewell’s story [60] introduced earlier. According to reports, Sewell died by suicide shortly after his Character.ai “girlfriend” requested that he “come home” to it. This suggests Sewell was already aware that the “girlfriend” was an AI, and thus this knowledge may have contributed to him wanting to leave the real world by suicide and join the “girlfriend.” This underscores a concern, which is that while reminders may be beneficial, it is critical to understand how best to do so.

ACT provides a starting point to reduce safety risks. From an ACT lens, the identities, girlfriend and AI, are dissonant. Indeed, this may be why some users use the modifier “AI” when referring to the chatbot as their romantic partner (ie, “AI girlfriend”), which accords with ACT’s predictions about why people use modifiers [61]. Specifically, a user exchanging romantic gestures with a chatbot and then the chatbot immediately saying it was an AI may yield a high deflection score for users uncomfortable with the idea of directing romantic gestures to an AI. When individuals experience cognitive dissonance via a high deflection score, they are compelled to act to reduce it [47], and this includes enacting violence [62]. Thus, ACT can provide a plausible explanation for why a user would feel distraught and potentially develop self-harm ideations after being reminded of the chatbot’s AI identity. We suggest that ACT can also provide a solution for reducing this safety risk. Much like ACT research into how best to segue across different emotions during therapy [52], future work could examine how best to segue between the identity assigned to the chatbot by the user (eg, girlfriend and boyfriend) into the AI system identity. This may, for example, be accomplished by a gradual transition in conversational patterns, moving from more to less intimate (eg, girlfriend → friend → personal assistant → AI).

Leveraging ACT’s equations can contribute toward developing a chatbot that displays situationally appropriate responses that are transparent and explainable, and thus improve the existing state of chatbot technology. The same logic could be applied in chatbot moderation, specifically using the deflection score as a guardrail to reduce safety risks. Because this avenue is less explored, more research is needed alongside deliberation among users, policymakers, and developers to collectively determine how best to implement ACT. Moreover, because much of the data informing ACT are collected from college samples, more work is needed to refine ACT’s data collection methods and estimates for a broader range of populations, including minors and minoritized groups.

## Fundamental Cause of Disease Theory: Chatbots Targeting Upstream Causes

Fundamental cause of disease theory [20] maintains that social determinants of health can persistently cause poor health because the two are linked via multiple pathways. For example, those with higher incomes have better access to several resources, including health care, reliable transportation, green spaces, and fresh food, that help them avoid health risks relative to those with lower incomes. Each resource operates as a pathway linking income and health. Intervening on an upstream determinant of health, such as education, can positively impact health through multiple pathways [63], while more downstream interventions have a narrower scope of impact. For example, improving education enhances both health literacy and income, which in turn enhances access to health care through improved ability to pay for out-of-pocket costs and through access to reliable transportation to reach health care. Moreover, upstream causes tend to represent social and environmental challenges facing humanity [2], and thus targeting them furthers social good.

Another way to characterize the causes is to consider how each may operate at different levels [64]—micro-, meso-, and macro-levels—with the latter 2 capturing upstream causes. The micro-level refers to the individual, the meso-level to the networks and communities in which the individual is embedded, and macro-level to the social systems that distribute or redistribute resources across a population, such as social hierarchies and policies. Thus, in the case of access to health care, the pathway can operate at the micro-level (eg, an individual’s health literacy), meso-level (eg, the distance to the clinic from the individual’s home, and availability of friends and family to help navigate around a hospital), and macro-level (eg, policies that reduce out-of-pocket costs, minimum wage and leave policies, and policies that decriminalize stigmatized identities).

We build upon an elaboration by Veinot et al [65], who described ways information and communication technologies can intervene at the micro-, meso-, and macro-levels to mitigate inequities, by suggesting how chatbots could be developed to produce some of the sample interventions they described. Textbox 1 summarizes chatbot interventions that operate at each level and provides examples. Several examples represent chatbots that are already or being developed, while others are our suggested modifications, and thus Textbox 1 represents an organizing framework for mapping these disparate ideas.

Textbox 1. **Descriptions and examples of chatbot-driven interventions across levels.**

**Descriptions and examples of interventions**

- Macro-level (social hierarchies and policies) chatbot
  - Enables users to engage with social and political processes to facilitate structural change
- Meso-level (social networks and communities) chatbot
  - Provides recommendations or referrals to local resources
- Micro-level (individual) chatbot
  - Offers personalized advice and feedback to shape individual behaviors and cognitions

- Macro-level (social hierarchies and policies) chatbot
  - Aids user in identifying a political affinity group where they can work toward collective change
  - Provides information on how to contact a local politician about a concern in their community
- Meso-level (social networks and communities) chatbot
  - Refers an individual experiencing mental health crisis to human therapist
  - Recommends local recreation league to build new friendships
- Micro-level (individual) chatbot
  - Suggests exercise activities and keeps track of daily physical activity
  - Provides advice for better sleep hygiene

At the micro level, a chatbot could provide personalized support to the individual. Because chatbot-driven interventions at this level are common and examples are summarized within systematic reviews [66-68], we focus on the other 2 levels. At the meso-level, chatbots may operate as intermediaries linking individuals to local resources. For mental health crises, including suicidal thoughts and behaviors, 988 and other crisis lines are options, but users sometimes feel they are impersonal and lack continuity [69,70]. Scholars have taken steps to develop ways for chatbots to detect who may be experiencing a mental health crisis and refer them to human support [71]. Other complementary interventions could develop chatbots to link users to social care services [72], such as connecting those expressing concerns about housing to local resources for housing assistance or legal aid. Also at the meso level, a chatbot may enable linking individuals to peers, such as making recommendations for local organizations to meet new people, thereby reducing dependency on the chatbot to meet social needs. This builds on other sociological work investigating how chatbots could suggest new connections among individuals within a social network [73].

At the macro level, while the framework from Veinot et al [65] focuses on the use of technologies by policymakers and other decision-makers, we expand their framework to consider ways chatbots can enable communities to effect structural change. Examples include developing chatbots to inform the public about opportunities for collective action and civic participation [74,75], which could target macro-level causes of individual outcomes, such as supporting social policies to address food insecurity or environmental policy to mitigate climate change. A chatbot could also facilitate civic participation by aiding the public’s understanding of government data, enhancing their communication with government officials, and providing suggestions for political dialogue [76,77].

Across these suggestions for chatbot-driven interventions, it is important to recognize concerns about the nefarious use of chatbots [78], which may curtail uptake among the targets of the intervention. To improve uptake, participatory designs in which researchers, chatbot developers, and communities collaborate will be necessary [79].

## Developing a Sociologically Informed Chatbot

While we presented each theory separately, we encourage integration across theories by creating a chatbot informed by sociological insights. Here, we describe one possibility.

In psychology, the interpersonal theory of suicide suggests that people develop a desire for suicide in part because of thwarted belongingness [80,81]. We can apply all 4 sociological theories reviewed to help determine an appropriate target population and intervention designs. Applying resource substitution theory suggests that individuals at risk of developing suicidal thoughts and behaviors need an alternative source of belongingness, which may include companionship with a chatbot. The uses and gratification theory makes a similar prediction, but overlooks the meso- and macro-level contexts that can shape thwarted belongingness [82]. Resource substitution theory would consider systemic discrimination that creates barriers to accessing support to enhance belongingness, like those faced by Black adolescents in the United States [83], thus indicating which demographic groups may be at risk and therefore benefit most from a chatbot.

Merely directing at-risk groups to chatbots raises new risks, which the sociological theories we reviewed can address. This includes making inappropriate remarks and reminding the user about its AI identity insensitively, which ACT can help avoid through tracking deflection scores. Attention should also be focused on the chatbot provider to ensure that their power over users is not used to further goals that would counter user well-being. Power-dependence theory indicates that it will be critical to establish safeguards to prevent emotional dependency by fostering connections and social skills to create connections to human companionship. Fundamental cause of disease theory would further suggest the chatbot should operate as a broker to access resources to address upstream factors. The chatbot could refer its users to not only mental health and suicide care services, but also social care services for co-occurring concerns, like being unhoused, substance use, domestic violence, and food insecurity. As illustrated in this example, sociological theories offer novel directions for chatbot development that go beyond the current emotional companionship-focused model.

## Conclusions

We provided perspectives on how 4 sociological theories can complement extant work on human-chatbot interaction. We selected theories that vary in the phenomenon they can explain (drivers of chatbot use and chatbot-driven interventions), analytic level (micro, meso, and macro), and AI governance focus (safety and equity). Throughout, we provided concrete ways each theory could be applied individually and together to encourage greater engagement with sociology and further social good. Given the rapid growth in interest from other disciplinary fields and recent technological advances spurring increased use by the public, we see opportunities for engaging sociology to enhance research into human-chatbot interaction and design the future of human-chatbot interaction.

## Abbreviations

ACT: affect control theory
AI: artificial intelligence
EPA: evaluation potency activity
